# Prevalence, Species Distribution and Resistance of Candidemia in Pediatric and Adult Patients in a Northeast Italy University Hospital

**DOI:** 10.3390/jof10100707

**Published:** 2024-10-10

**Authors:** Silvia Meneghello, Giulia Bernabè, Giuseppe Di Pietra, Sarah Di Sopra, Claudia Del Vecchio, Anna Maria Cattelan, Ignazio Castagliuolo, Paola Brun

**Affiliations:** 1 Microbiology and Virology Unit, Padova University Hospital, Via Giustiniani 2, 35128 Padua, Italy; silvia.meneghello92@gmail.com (S.M.); giuseppe.dipietra@studenti.unipd.it (G.D.P.); sarah.disopra@studenti.unipd.it (S.D.S.); claudia.delvecchio@unipd.it (C.D.V.); ignazio.castagliuolo@unipd.it (I.C.); 2 Department of Molecular Medicine, Section of Microbiology, University of Padova, Via A. Gabelli 63, 35127 Padova, Italy; giulia.bernabe@unipd.it (G.B.); annamaria.cattelan@unipd.it (A.M.C.); 3 Infectious Diseases Unit, Padova University Hospital, Via Giustiniani 2, 35128 Padua, Italy

**Keywords:** bloodstream infections, *Candida*, resistance, non-*albicans Candida*, uncommon *Candida*, epidemiology

## Abstract

Candidemia and invasive candidiasis (IC) are causes of morbidity and mortality in healthcare settings, with notable differences between children and adults. Understanding the species distribution and antimicrobial susceptibility profiles of clinical isolates can guide empiric therapy in patients at risk of IC. This study investigated the incidence and antifungal susceptibility patterns of yeasts involved in IC in pediatric and adult patients from 2019 to 2023. The average incidence of IC was 0.715 per 1000 patients, increasing over the study period; infants had the highest incidence rates. Over half of the IC episodes occurred in intensive care units (ICUs). Non-*albicans Candida* (NAC) species represented the most frequently isolated species in adults and children (55.96% and 50.0%, respectively), with the prevalence of *C. parapsilosis* (26.45% and 14.7%, respectively), *N. glabratus* (14.97% and 8.82%, respectively) and *C. tropicalis* (4.36% and 2.94%, respectively). *C. lusitaniae* was identified in 14.7% of pediatric IC cases. In NAC species, antifungal resistance has also increased over the five years of the study: 69.12% were resistant to azoles and 7.35% were resistant to micafungin. Resistance was higher in pediatric patients. Our study highlights differences in IC characteristics between pediatric and adult populations and emphasizes the importance of targeted antifungal stewardship in ICU patients with NAC invasive infections.

## 1. Introduction

*Candida* species are members of the commensal microbial community, as they are detected on the skin and mucosal surfaces of 50–70% of healthy humans [1,2]. However, *Candida* spp. can become pathogenic, causing various infections in both immunocompetent and immunocompromised subjects [3,4]. Long-term use of broad-spectrum antibiotics, alterations in the integrity of the mucocutaneous barriers (i.e., indwelling central venous catheter, gastrointestinal surgery, intestinal mucositis), chemotherapy-induced neutropenia or corticosteroid therapy perturb mucosal microbiota and weaken the host immunity, thus facilitating the transition from commensalism to opportunism in *Candida* spp. [5]. In fungi, opportunism is associated with the expression of virulence factors such as adhesion to medical devices, hyphal penetration and invasion and vascular dissemination [5,6,7], leading to invasive candidiasis (IC). IC refers to bloodstream infections with *Candida* spp. (namely, candidemia) and deep-seated infections such as intra-abdominal abscess, peritonitis or osteomyelitis, with or without candidemia [5].

IC is a critical healthcare-associated fungal infection with increasing worldwide incidence [8]. Candidemia accounts for 22% of cases of bloodstream infections [5,9], ranking as the fourth most common bloodstream infection in intensive care units [5]. The incidence of candidemia is age-related, with maximum rates observed in pediatric and elderly patients [10,11]. In the United States, candidemia is recognized as the second most common cause of healthcare-associated bloodstream infection [4,8]. In Europe, the estimated pooled annual incidence rate of IC is 3.88 per 100,000 people, with 7.07 episodes per 1000 intensive care unit admissions [12,13]. *C. albicans*, *C. parapsilosis*, *C. tropicalis*, *Nakaseomyces glabratus* (formerly *C. glabrata*) and *Pichia kudriavzevii* (formerly *C. krusei*) are responsible for more than 90% of candidemia cases [14]. Indeed, recent epidemiological studies have reported an increased relative prevalence of non-*albicans Candida* (NAC) species in Europe [13,15,16]. Over the last two decades, episodes of candidemia have revealed increased incidence also in Italy. A one-year prospective survey of candidemia from 34 Italian departments of clinical microbiology revealed a threefold increase in incidence compared to a study performed ten years earlier [17,18]. Brescini L. and colleagues reported a 2.5/1000 hospital admission cumulative incidence of candidemia, significantly increasing over eight years of the survey [19]. All Italian studies reported a high thirty-day mortality rate (19.8–28%) that directly correlated with neutropenia, pneumoniae and infection with *C. albicans* and was not influenced by the type of antifungal therapy [12,17,20]. A rise in antifungal resistance was recently reported with the emergence of fluconazole- and echinocandin-resistant *Candida* strains [20]. Prospective and retrospective studies at Policlinico San Martino Teaching Hospital in Genoa, Italy, revealed fluconazole resistance in 33% of *C. parapsilosis* isolates [21]. Echinocandin-resistant *N. glabratus* and multidrug-resistant *C. auris* have been also reported [22].

Candidemia is more frequent in children than adults, with the highest rates of invasive fungal infections diagnosed in preterm infants and infants under one [23,24]. Compared with adults, candidemia in pediatric patients is associated with better clinical outcomes, minimal antifungal resistance and low mortality rates but higher inpatient costs [23]. Moreover, pediatric and adult populations differ in terms of the prevalence of *Candida* species even in the same geographic areas [20,25]. Early diagnosis is challenging in children because of the nonspecific symptoms of candidemia and low sensitivity of blood cultures [26]. Nonetheless, over recent decades, antimicrobial stewardship practices have decreased the incidence of IC in children in the United States [27]. Therefore, the surveillance of local species distribution and antifungal susceptibility in pediatric and adult patients suffering from IC is mandatory in supporting the rational choice of empiric therapy and antimicrobial stewardship. In this study, we aimed to investigate the epidemiology of IC in pediatric and adult patients to determine the local species distribution and antimicrobial susceptibility during five years of surveillance at the Padova University Hospital.

## 2. Materials and Methods

### 2.1. Data Retrieval

A retrospective study of invasive candidiasis (IC) in patients admitted at the Padova University Hospital between January 2019 and December 2023 was conducted to evaluate the incidence, species distribution and antifungal susceptibility. The study received approval from the Data Management Unit of the Padova University Hospital (approval n. 2772 on 30 December 2022) in accordance with the Ethical Code reported in DGR n. 1633 on 19 December 2022. The Data Management Unit of the Padova University Hospital also carried out data anonymization. Episodes of IC were defined as positive blood cultures for *Candida* species. Each patient was considered only once for cases involving multiple isolations of the same *Candida* species. During the study period, all IC-positive samples were included in the study with no exclusion criteria.

### 2.2. Candida spp. Identification and Antifungal Susceptibility Test

*Candida* species identification was performed during routine work at the Microbiology and Virology Unit of the Padova University Hospital using standard microbiological procedures. No changes in the laboratory procedures were introduced during the five years of the study. Blood samples were inoculated into aerobic culture bottles incubated into a BACT/ALERT^®^ VIRTUO^®^ Microbial Detector System (Biomerieux Inc., Firenze, Italy). Positive samples with fungal cells visible on Gram stain examination were plated on Sabouraud Dextrose Emmons Agar (SGC2, Biomerieux Inc.). Colonies were identified using the Matrix-Assisted Laser Desorption/Ionization Time of Flight (MALDI TOF) Vitek^®^ MS (Biomerieux Inc.). Data were elaborated by Myla software v.1 based on the comparison of mass spectra in Knowledge Base VITEK^®^ MS v3.2. Results were scored according to the manufacturer’s criteria. Specifically, the identification at genus level was considered precise with a value > 95.9%. Antifungal susceptibility was tested using the Sensititre™ ARIS™ 2X ID/AST System (Thermo Fisher Scientific, Milan, Italy) using Sensititre YeastOne™ YO10 AST plates (Thermo Fisher Scientific). The breakpoints of MIC values (Table 1) were interpreted according to the criteria of the Clinical and Laboratory Standards Institute (CLSI) [28,29].

### 2.3. Statistical Analysis

Descriptive statistics was used to summarize data, including the distribution of *Candida* spp. among age groups, years of study and hospital wards. Variables were evaluated using the Chi-square test or the two-tailed Fisher exact test. We reported continuous variables using median and fist-third quartile values, and we compared groups with Student’s *t* test (two groups) or ANOVA (three or more groups) when data were normally distributed or with the Mann–Whitney test (two groups) or Kruskal–Wallis test (three or more groups) when data were not normally distributed. A *p*-value equal to or less than 0.05 was considered statistically significant. All analyses were performed using SPSS Statistics version 20.0 (IBM Corp., New York, NY, USA).

## 3. Results

### 3.1. Incidence of Invasive Candidiasis

**Figure 1:**
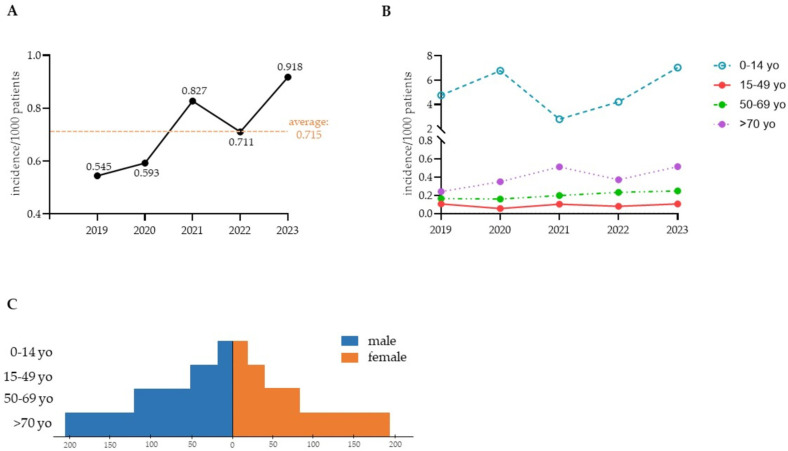
The overall incidence of invasive candidiasis (IC) episodes per 1000 patients admitted to Padova University Hospital between 2019 and 2023. For each year, the incidence value is reported; the dotted orange line indicates the calculated average incidence (**A**). The annual incidence of IC by age group; yo: years old (**B**). Gender distribution of patients with IC; yo: years old (**C**).

Between January 2019 and December 2023, 722 episodes of invasive candidiasis (IC) in 705 patients were identified at Padova University Hospital. Seventeen patients were diagnosed with more than one fungal species, as better described later (paragraph 3.2). The average incidence of IC was 0.715 per 1000 patients (Figure 1A). In the pediatric population (ages 0–14), the five-year average incidence was 5.067 per 1000 patients (Figure 1B). In contrast, patients 15–99 years of age had an average incidence of 0.686 per 1000 patients (Figure 1B). As shown in Figure 1A, in the general population, the incidence of IC increased from 0.545/1000 patients in 2019 to 0.918/1000 patients in 2023. An analysis of age-specific trends (Figure 1B) indicated that the pediatric population and elderly patients (over 70 years old (yo)) experienced the highest increases in incidence rate ratio ((IRR) 1.48, 95% confidence interval (CI) 1.38–1.69, *p* < 0.02 in pediatric patients; IRR 2.13, 95% CI 2.09–2.18, *p* = 0.05 in elderly patients). Patients aged 15–49 yo and 50–69 yo did not show significant changes in the incidence rates over time (IRR 0.99, CI 0.92–1.08; IRR 1.3, CI 0.97–1.19, respectively) (Figure 1B).

Among pediatric patients suffering from IC, 52.9% were male; in the youth group (15–49 yo), 60% were male; among the middle-aged patients (50–69 yo), 62.8% were male; in the elderly population (>70 yo), 53.11% were male (Figure 1C).

### 3.2. Species Distribution

**Figure 2:**
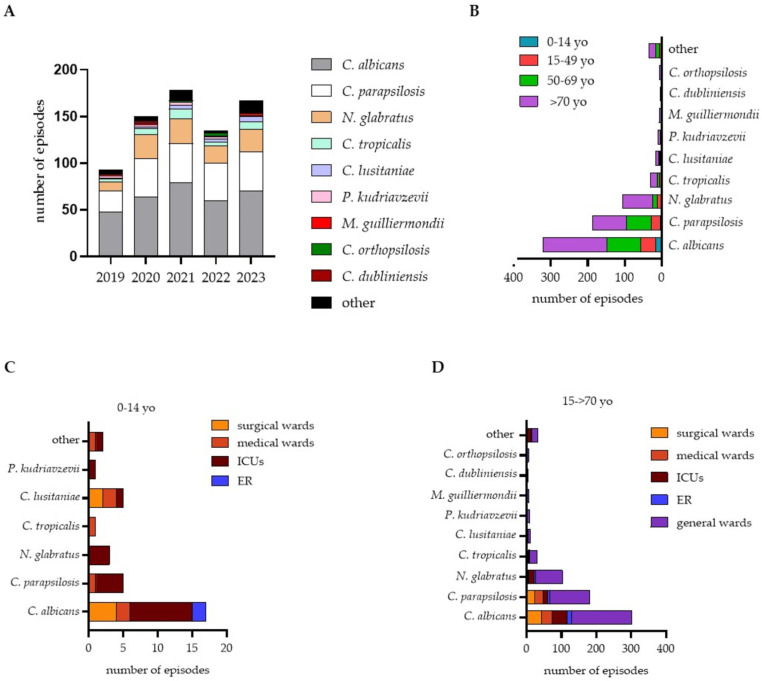
Distribution of clinical isolates of invasive candidiasis (IC). Episodes of IC were tallied by species and year and graphed (**A**). Episodes of IC were tallied by species and age of patients and graphed; yo: years old (**B**). Episodes of IC in the pediatric population (0–14 yo) were tallied by species and wards of the first identification; ICUs: intensive care units; ER: emergency room (**C**). Episodes of IC in patients aged 15–>70 years were tallied by species and wards of the first identification; general wards: long-stay units (**D**).

The yearly distribution of *Candida* species causing IC is reported in Figure 2A. Overall, *C. albicans* was the predominant species (44.32%), followed by *C. parapsilosis* (25.90%), *N. glabratus* (14.68%) and *C. tropicalis* (4.29%). Other identified *Candida* species were *C. lusitaniae* (2.22%), *P. kudriavzevii* (1.38%), *Meyerozyma guilliermondii* (formerly *C. guilliermondii*, 0.97%), *C. orthopsilosis* (0.83%) and *C. dubliniensis* (0.55%). In 17 patients with IC (2.41%), infection was caused by more than one *Candida* spp.; most commonly, *C. albicans* and *C. parapsilosis* were simultaneously isolated (52.94% of the multiple infections) followed by the combination of *C. albicans* with *N. glabratus* (23.52%), *C. parapsilosis* with *C. orthopsilosis* (11.76%) and one case of *N. glabratus* co-isolated with *C. tropicalis*. In one patient, we also observed *C. albicans* associated with *Papiliotrema laurentii*.

Compared to 2019, from 2020 onwards, the relative incidences of *C. parapsilosis* and *N. glabratus* have increased by 1.86 fold (95% CI 8.12–11.78, *p* < 0.05) and 2.6 fold (95% CI 5.74–7.20, *p* < 0.05), respectively. Notably, the incidence of less frequently reported *Candida* species, categorized as “other” in Figure 2A, has risen by 2.4 fold (95% CI 2.64–5.05, *p* < 0.05) in five years. In this group, we primarily identified *Debaryomyces hansenii* (formerly *C. famata*), *C. metapsilosis*, *Yarrowia lipolytica* (formerly *C. lipolytica*) and *C. pelliculosa*. We observed that the relative increase in the incidence of these *Candida* species affected patients across all age groups (Figure 2B). At the same, *C. albicans* infections occurred in all age groups. On the contrary, patients with *N. glabratus* and *C. parapsilosis* infections were significantly older (Table 2). Specifically, when normalized to the cases of IC per age group, infections with *C. parapsilosis* were predominantly identified in young (15–49 yo) and middle-aged (50–69 yo) patients (Figure 2B). *C. lusitaniae* was more frequently identified in pediatric patients than in other age groups (*p* < 0.05, Table 2). In the pediatric population, most cases of IC were identified in intensive care units (ICUs), followed by surgical wards and medical (gastroenterology and cardiology) wards (Figure 2C). In contrast, in patients over 15, IC was primarily diagnosed in long-stay wards and burn and transplant units, followed by ICUs and surgical wards (Figure 2D).

### 3.3. Antifungal Resistance

**Figure 3:**
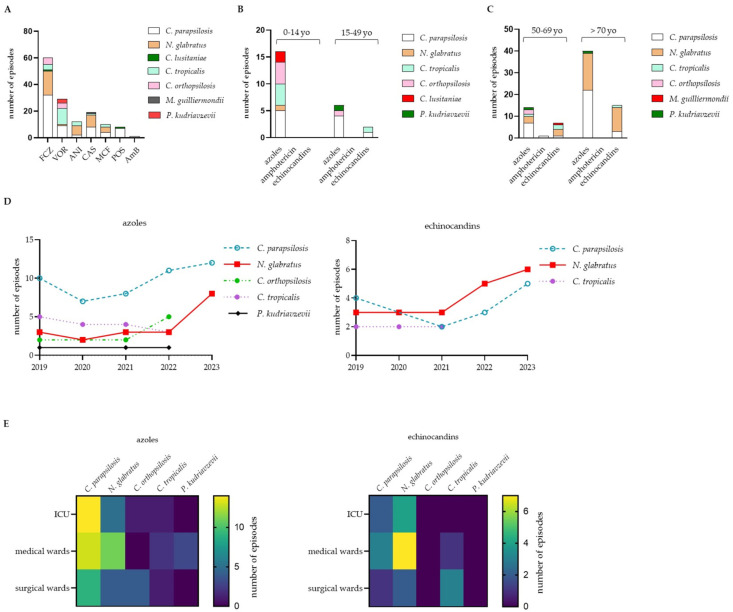
Antifungal resistance in clinical isolates of invasive candidiasis (IC). *Candida* spp. were clustered for resistance; FCZ: Fluconazole; VOR: Voriconazole; ANI: Anidulafungin; CAS: Caspofungin; MCF: Micafungin; POS: Posaconazole; AmB: Amphotericin B (**A**). *Candida* spp. were clustered for resistance to azoles (FCZ, VOR, POS), amphotericin (AmB) or echinocandins (ANI, CAS, MCF) and age of the patients; yo: years old (**B**,**C**). *Candida* spp. were clustered for resistance to azoles (left panel) or echinocandins (right panel) and year of isolation (**D**). Episodes of *Candida* spp. were clustered for resistance to azoles (left panel) or echinocandins (right panel) and the ward of the first isolation; ICU: intensive care unit (**E**).

Susceptibility tests were performed in all clinical isolates for Amphotericin B (AmB), Anidulafungin (ANI), Caspofungin (CAS), Fluconazole (FCZ), Micafungin (MCF), Posaconazole (POS), Voriconazole (VOR) and Itraconazole (ITR). All isolates were susceptible to ITR. The tested antifungal drugs showed excellent activity against *C. albicans* and *C. dubliniensis*. In five years, we identified 77 (10.66%) episodes of IC with resistance to at least one drug. Among the clinical isolates, resistance to FCZ was the most prevalent, occurring in 32 cases of *C. parapsilosis* infections, 18 cases of *N. glabratus* and 5 cases of *C. orthopsilosis* (Figure 3A). *C. parapsilosis* isolates were also resistant to VOR, CAS, POS, MCF and ANI; *N. glabratus* and *C. tropicalis* isolates were resistant to VOR, CAS, MCF and ANI; and *C. orthopsilosis* also reported resistance to VOR. CAS resistance was noted in one *M. guilliermondii* isolate (Figure 3A). Resistance to two or more drugs was reported in 39 isolates (50.64% of resistant isolates).

The higher incidence of resistant isolates was described in the pediatric population (Table 3). Among the pediatric population, 26.47% of the isolates reported resistance to one or more azoles (e.g., FCZ, VOR and POS; Table 3). Resistance to FCZ plus VOR was identified in two out of three *C. parapsilosis*-resistant strains and in two out of two *C. orthopsilosis* and *C. tropicalis* isolates. We also identified one *C. lusitaniae* isolate that was simultaneously resistant to FCZ and POS.

In patients aged 0–14, *C. parapsilosis* (33.33%), *C. orthopsilosis* (22.22%) and *C. tropicalis* (22.22%) were the most prevalent resistant strains (Figure 3B). No resistance to echinocandins was reported in the isolates obtained from the pediatric population (Figure 3B, Table 3). The percentage of resistant isolates in the youth group (15–49 yo) was 9.09%. The strains resistant to one or more azoles (e.g., FCZ and POS) were *C. parapsilosis* (50%), *C. orthopsilosis* (12.5%) and *P. kudriavzevii* (12.5%). We identified one isolate of *C. tropicalis* resistant to ANI and one *C. parapsilosis* isolate resistant to ANI and CAS (Figure 3B). In the middle-aged group (50–69 yo), the percentage of resistant isolates was 9.04%; resistance to azoles was identified in *C. parapsilosis* (38.88%), *C. orthopsilosis* (11.11%), *C. tropicalis* and *N. glabratus* (Figure 3C). *N. glabratus* isolates that were resistant to FCZ also reported resistance to ANI. One *C. parapsilosis* isolate reported resistance to all three tested echinocandins. We also identified one *C. parapsilosis* isolate resistant to AmB (Figure 3C). In the elderly patients, 10.47% of isolates had resistance. Almost all *C. parpasilosis*-resistant isolates (95.65%) were unresponsive to azoles, whereas one isolate reported resistance to MCF only (Figure 3C). Other resistant isolates were *P. kudriavzevii* (resistance to azoles), *C. tropicalis* (resistance to echinocandins) and *N. glabratus*. Resistance to echinocandins reached almost 62% in *N. glabratus* (Figure 3C).

Between 2019 and 2021, the incidence of isolates resistant to azoles and/or echinocandins was almost constant. Between January 2022 and December 2023, we registered a worrisome increase in the incidence of *N. glabratus* isolates resistant to echinocandins (14.29, 95% CI 12.32–18.08, *p* < 0.05) and, starting from January 2023, also in *N. glabratus* isolates resistant to azoles (26.42, 95% CI 20.88–27.12, *p* < 0.02; Figure 3D). The resistance in *C. orthopsilosis*, *P. kudriavzevii* and *C. tropicalis* almost disappeared in 2023 (Figure 3D). Table 4 reports the MIC distribution of the resistant *Candida* spp. Notably, an overall increment, although not significant, in fluconazole and echinocandins MIC for *C. parapsilosis* and *N. glabratus* was observed over the five years of the study (Table 4). Of concern is the increasing resistance of *C. tropicalis* to VOR, ANI and MCF (Table 4).

As reported in Figure 3E, most of *C. parapsilosis* and *N. glabratus* strains resistant to azoles were isolated in the ICU and medical wards, whereas *N. glabratus* isolates resistant to echinocandins were primarily identified in medical wards. Resistant *C. lusitaniae* were also identified in medical wards.

## 4. Discussion

Several studies aimed to investigate the epidemiology of invasive candidiasis, species distribution and antifungal susceptibility profiles [11,12,13,22,30]. In this study, we reviewed these aspects in adult and pediatric populations over five years to provide an integrated analysis of the trends of invasive fungal infections in a Northeastern Italy University Hospital.

Our findings indicate a 55.69% increase in the overall incidence rate of IC over five years (Figure 1A), which contrasts with trends reported in the USA [31,32] but is in accordance with global [4], European and Italian epidemiological data [30,33]. The incidence of IC is affected by various factors, including geographical location, the healthcare system, patient demographics and environmental conditions [34]. The Padova University Hospital and the Microbiological and Virological Unit serve a large population in the Padua metropolitan area in Northeastern Italy, encompassing a wide variety of clinical cases. Additionally, our study assessed the incidence of IC across a large population, including patients from infancy to over 70 years of age (Figure 1C). Age-specific trends (Figure 1B) showed the highest IC increase in the pediatric population, differing from data reported in the USA, where IC in children under four decreased by 62% over 11 years [31].

The upsurge in antifungal resistance described in our study (Figure 3) cannot explain the increase in the incidence of IC since antifungal prophylaxis is routinely limited to patients with cancer, transplantation or hematological malignancies [35]. Multiple factors are involved in the rise in IC incidence. Advances in medicine have improved survival rates for patients with severe illnesses, thereby highlighting new risk groups for IC, such as those with weakened immune systems and those undergoing invasive medical procedures [36,37]. In addition, as *Candida* spp. are common components of the gut and skin microbiota, endogenous sources of candidemia have been suggested [34,38,39]. This hypothesis is based on the observation that disruptions in host defenses, corticosteroid treatment, antibiotic usage and immunosuppression are associated with invasive fungal infections in patients hospitalized with SARS-CoV-2 or influenza [40,41,42].

In addition to the increased incidence of IC, global reports underline the emergence of new fungal pathogens with a shift to non-*albicans Candida* (NAC) species involved in invasive infections [36]. In accordance with the SENTRY Surveillance Program [43], in our study, *C. albicans* was identified in 44.32% of episodes of IC, whereas NAC species account for 55.68% of the cases (Figure 2, Table 2). Our data are in line with previous Italian reports, ranking *C. albicans* as the most frequently isolated species (50–62%), followed by *C. parapsilosis* (17–25%) and *N. glabratus* (7–13%) [17,19,21]. Other reported species were *C. tropicalis*, *P. kudriavzevii*, *M. guilliermondii*, *C. lusitaniae*, *C. dubliniensis*, *C. famata*, *C. kefyr*, *C. norvegensis* and *C. etchellsii* [17,19,21]. An outbreak of *C. auris* has been reported in the Liguria region since 2019 [44]. In our study, 13 out of 705 patients with IC were simultaneously infected with *C. albicans* and an NAC species. Even if *C. albicans* was the most frequently identified yeast also in the pediatric population (Figure 2B), a higher proportion of IC caused by *C. lusitaniae* was observed among patients 0–14 years old; children admitted to the ICU presented the highest rates of NAC species (Figure 2C). No cases of *C. auris*, reported in the priority list of fungal pathogens by the WHO [36], were retrieved in our study. On the contrary, we observed an increasing trend in usually rare *Candida* spp. such as *C. lusitaniae* and *M. guilliermondii* (Figure 3A). Advances in diagnostic technologies have enabled accurate identification of previously uncommon *Candida* species, which now pose new necessities in the surveillance of local epidemiology and antimicrobial susceptibility [45,46]. Indeed, most uncommon *Candida* species are intrinsically resistant or lack official antifungal susceptibility profiles. When available, susceptibility or resistance are inferred by comparing MIC values with common *Candida* species. As a result, the antifungal therapy may be less effective, leading to potential treatment failures, suboptimal outcomes or drug resistance in the emerging species [47].

Therapeutic options for IC are limited to four classes of antifungals: polyenes, 5-fluorocytosine, azoles and echinocandins. While azoles and echinocandins are generally effective, agricultural use of azole pesticides has contributed to resistance in environmental and clinical yeast isolates [48]. Unlike recent studies [19,25,30], we observed no fluconazole resistance in *C. albicans*, but resistance in NAC species increased over five years (Figure 3D, Table 2). Notably, micafungin resistance rose in *C. parapsilosis*, *N. glabratus* and *C. tropicalis* (Figure 3A, Table 2), affecting mainly patients aged 0–14 and >70 in the ICU (Figure 3C,E).

Our study has several limitations: it is a single-center, retrospective, observational study; due to the absence of a control group and limited accessibility to clinical data, we could not make any causality inferences regarding patient survival and treatments. The genetic features of isolated strains are currently under investigation and will be discussed in future studies; however, at the moment, the lack of information may lead to misdiagnoses for a few species. Finally, it should be noted that since IC can occur with negative blood culture [49], some data regarding species distribution and antifungal resistance may be imprecise.

In conclusion, in this study, we showed that infants and elderly patients admitted to ICU are at a higher risk of IC caused by NAC species. Moreover, we reported unique characteristics in species distribution and antimicrobial profile in pediatric IC. The observed differences between the pediatric and adult populations and trends between 2019 and 2023 point towards focused antifungal stewardship policies for patients admitted to the ICU and require further microbiological exploration.

## Figures and Tables

**Table 1:** Antifungal susceptibility interpreted according to CLSI breakpoints (CLSI M59-ED3 and M60-ED2).

*Candida* spp.	Antifungal Agent	MIC (µg/mL)		
		S	I/SDD	R
*C. parapsilosis*	FCZ	≤2	4	≥8
	VOR	≤0.12	0.25–0.5	≥1
	POS	0.25 *	1	1
	ANI	≤2	4	≥8
	MCF	≤2	4	≥8
	CAS	≤2	4	≥8
*N. glabratus*	FCZ	-	≤32	≥64
	VOR	0.25 *		
	ANI	≤0.12	0.25	≥0.5
	MCF	≤0.06	0.12	≥0.25
	CAS	≤0.12	0.25	≥0.5
*C. tropicalis*	FCZ	≤2	4	≥8
	VOR	≤0.12	0.25–0.5	≥1
	ANI	≤0.25	0.5	≥1
	MCF	≤0.25	0.5	≥1
*P. kudriavzevii*	VOR	≤0.5	1	≥2
*C. orthopsilosis*	FCZ	2 *		
	VOR	0.125 *		
*C. lusitaniae*	FCZ	1 *		
	POS	0.06 *		
*M. guilliermondii*	CAS	≤2	4	≥8

MIC: minimal inhibitory concentration; S: susceptible; I: intermediate; SSD: susceptible-dose dependent; R: resistant. * ECV (µg/mL), epidemiological cutoff values from CLSI M59-ED3 were considered when MIC values were unavailable in CLSI M60-ED2.

**Table 2:** Species distribution among the studied populations. Patients were grouped into pediatric (0–14 yo) and adults (15–>70 yo).

*Candida* spp.	0–14 yo		15–>70 yo	
*C. albicans*	17 (50%)		303 (44.04%)	
*C. parapsilosis*	5 (14.70%)	NAC 17 (50%)	182 (26.45%)	NAC 385 (55.96%)
*N. glabratus*	3 (8.82%)		103 (14.97%)	
*C. tropicalis*	1 (2.94%)		30 (4.36%)	
*C. lusitaniae*	5 (14.70%)		11 (1.60%)	
*P. kudriavzevii*	1 (2.94%)		9 (1.31%)	
*M. guilliermondii*	0		7 (1.02%)	
*C. orthopsilosis*	0		4 (0.58%)	
*C. dubliniensis*	0		6 (0.87%)	
other	2 (5.88%)		33 (4.80%)	

Data are reported as numbers and (percentages). Percentages were calculated over the total number of episodes of *Candida* spp. infection per age group. yo: years old; NAC: non-*albicans Candida* spp.

**Table 3:** Resistance in *Candida* spp. among the studied populations. Patients were grouped into pediatric (0–14 yo) and adults (15–>70 yo). Multiple resistances are reported.

*Candida* spp.	Antifungal Agent	0–14 yo	15–>70 yo
*C. parapsilosis* total n: 62	FCZ	3 (18.75%)	29 (23.58%)
	VOR	2 (12.5%)	7 (5.69%)
	POS	1 (6.25%)	6 (4.88%)
	ANI	0	2 (1.63%)
	MCF	0	4 (3.25%)
	CAS	0	8 (6.50%)
*N. glabratus* total n: 39	FCZ	0	18 (14.63%)
	VOR	1 (6.25%)	0
	ANI	0	7 (5.69%)
	MCF	0	4 (3.25%)
	CAS	0	9 (7.32%)
*C. tropicalis* total n: 22	FCZ	2 (12.5%)	3 (2.44%)
	VOR	2 (12.5%)	10 (8.13%)
	ANI	0	3 (2.44%)
	MCF	0	2 (1.63%)
*P. kudriavzevii* total n: 0	VOR	0	4 (3.25%)
*C. orthopsilosis* total n: 9	FCZ	1 (6.25%)	4 (3.25%)
	VOR	1 (6.25%)	3 (2.44%)
*C. lusitaniae* total n: 3	FCZ	2 (12.5%)	0
	POS	1 (6.25%)	0

Data are reported as numbers and (percentages). Percentages were calculated over the total number of episodes of *Candida* spp. infection per age group. yo: years old.

**Table 4:** Antifungal MIC and *Candida* spp. Only species with more than one episode of resistance were reported.

*Candida* spp.	Antifungal Agent	MIC (µg/mL)				
		2019	2020	2021	2022	2023
*C. parapsilosis*	FCZ	8 (8–8)	16 (12–96)	64 (50–64)	32 (16–64)	64 (16–64)
	VOR	0.5 (0.5–0.5)	0.5 (0.5–0.5)	0.5 (0.5–0.5)	0.5 (0.5–0.5)	0.5 (0.5–0.5)
	POS	0.31 (0.12–1.625)	0.12 (0.12–0.12)	0.12 (0.12–0.12)	-	0.12 (0.12–0.12)
	ANI	-	-	-	-	1 (1–1)
	MCF	1 (1–1)	-	8 (8–8)	8 (8–8)	–
	CAS	6 (6–6)	-	6 (6–6)	6 (6–6)	6 (6–6)
*N. glabratus*	FCZ	8 (8–8)	144 (32–256)	128 (8–256)	256 (256–256)	192 (128–256)
	VOR	0.25 (0.25–0.25)	-	-	-	-
	ANI	0.5 (0.5–0.5)	0.5 (0.5–0.5)	–	8 (8–8)	8 (0.5–8)
	MCF	-	8 (8–8)	8 (8–8)	8 (8–8)	8 (8–8)
	CAS	8 (8–8)	8 (8–8)	8 (8–8)	8 (8–8)	8 (8–8)
*C. tropicalis*	FCZ	16 (16–16)	16 (16–16)	-	16 (16–16)	-
	VOR	0.25 (0.15–0.43)	0.25 (0.25–0.25)	0.25 (0.25–0.25)	1 (1–1)	-
	ANI	0.12 (0.12–0.12)	0.25 (0.25–0.25)	4 (4–4)	-	-
	MCF	-	2 (2–2)	2 (2–2)	-	-
*C. orthopsilosis*	FCZ	16 (16–16)	-	16 (16–16)	16 (16–16)	-
	VOR	0.5 (0.5–0.5)	-	0.5 (0.5–0.5)	0.5 (0.5–0.5)	-
*P. kudriavzevii*	VOR	32 (32–32)	-	32 (32–32)	32 (32–32)	-
*C. lusitaniae*	FCZ	-	-	-	2 (2–2)	-
	POS	-	-	-	0.06 (0.06–0.06)	-

MIC: minimum inhibitory concentration. MICs are reported as median and first–third quartile. FCZ: Fluconazole; VOR: Voriconazole; POS: Posaconazole; ANI: Anidulafungin; MCF: Micafungin; CAS: Caspofungin.

## Data Availability

The original contributions presented in the study are included in the article, further inquiries can be directed to the corresponding author.
